# Structural Cerebral Correlates of Perplexity: Exploring a Linguistic Marker in Cognitive Aging

**DOI:** 10.1002/pchj.70010

**Published:** 2025-03-14

**Authors:** Xingsong Wang, Christina J. Herold, Claudia Frankenberg, Ayimnisagul Ablimit, Tanja Schultz, Li Kong, Johannes Schröder

**Affiliations:** ^1^ School of Psychology Shanghai Normal University Shanghai China; ^2^ Section of Geriatric Psychiatry, Department of General Psychiatry University of Heidelberg Heidelberg Germany; ^3^ Cognitive Systems Lab Bremen University Bremen Germany

**Keywords:** cognitive aging, linguistic, mild cognitive impairment, MRI, perplexity

## Abstract

Language changes are among the earliest indicators of cognitive decline in aging. Perplexity, a linguistic measure derived from information theory that quantifies speech predictability, has emerged as a potential marker for detecting early cognitive changes. However, its underlying neural substrates remain unclear. This study investigated the structural brain correlates of perplexity in 38 elderly participants (26 cognitively healthy, 12 with mild cognitive impairment) using magnetic resonance imaging (MRI). Perplexity was computed automatically from autobiographical interviews using single‐word (1‐g) and word‐pair (2‐g) models. Voxel‐based morphometry analyses, adjusted for total intracranial volume, sex, and education, revealed distinct associations between perplexity measures and regional gray matter volume. Region‐of‐interest analyses confirmed significant positive correlations between 1‐g perplexity and left middle temporal gyrus volume as well as between 2‐g perplexity and left precuneus. These findings suggest that perplexity reflects both linguistic processing and autobiographical memory, as evidenced by its associations with language‐relevant temporal regions and memory‐related precuneus. This study provides initial insights into the neural basis of perplexity as a measure that captures both linguistic and content‐related aspects of language production in cognitive aging.

## 1 Introduction

Linguistic changes are consistently observed in individuals with Alzheimer's disease (AD) and its clinical precursor, mild cognitive impairment (MCI), which is associated with an increased risk of progression to AD (Schröder and Pantel 2011). While MCI is characterized by neuropsychological impairments that go beyond those typically observed during normal aging, these deficits are not (yet) comparable to the more severe losses associated with early AD.

Linguistic changes associated with MCI/AD manifest across several levels. At the surface level, reduced word fluency—particularly category fluency—and word‐finding difficulties emerge during the transition from healthy aging to AD (Andrejeva et al. 2016; Barth et al. 2005; Dos Santos et al. 2011; Lukatela et al. 1998; Rinehardt et al. 2014; Sutin et al. 2019). Additionally, studies report diminished lexical diversity and richness, likely driven by increased repetition of nouns and verbs (Berisha et al. 2015; Bucks et al. 2000; de Lira et al. 2011). These changes are often accompanied by a rise in speech pauses (López‐de‐Ipiña et al. 2013). At deeper linguistic levels, progressive disruptions in language integrity are evident even during the prodromal stages of AD. These include reduced syntactic complexity and impairments in semantic and lexical content (Ahmed et al. 2013). Ultimately, these changes culminate in a marked simplification of spoken language as AD advances.

Given the conceptualization of AD as a progressive neurodegenerative disorder that begins decades before the clinical manifestation (Lloret et al. 2019; Schröder et al. 2004), the identification of preceding cognitive markers is of special interest. Recent findings from our research indicated that verbal fluency deficits can predict subsequent cognitive deterioration (progression to MCI or AD) more than a decade prior to clinical diagnosis. This observation emerged from our analysis of 246 cognitively intact participants (mean age at baseline: 62.77 ± 0.91 years) who were followed as part of the Interdisciplinary Longitudinal Study on Adult Development and Aging (ILSE) (Frankenberg et al. 2021). Additionally, a study of 145 manually transcribed ILSE interviews by Wendelstein (2016) identified distinctive linguistic patterns in individuals with preclinical AD compared to healthy controls across a 12‐year period. These patterns were characterized by reduced lexical richness and the overproduction of pronouns and incomplete syntactic phrases.

Numerous studies have investigated linguistic and speech‐based markers of cognitive decline, reporting detection rates of 89% for AD and 82% for MCI when compared to healthy controls (de la Fuente Garcia et al. 2020; Petti et al. 2020). Linguistic analysis of clinical interviews has demonstrated significant potential in identifying prodromal manifestations of AD years before a clinical diagnosis (Wendelstein 2016). This underscores the value of computational approaches to speech analysis as diagnostic tools. Among these computational methods, perplexity—a measure derived from information theory—shows particular promise in quantifying speech predictability (for overview see: Hale et al. 2022; Jurafsky and Martin 2023). Perplexity captures both lexical and sequential aspects of language production through complementary language models (1‐g and 2‐g), providing insights into different levels of language complexity (Frankenberg et al. 2019; Weiner et al. 2017). Our group previously demonstrated that perplexity predicted the severity of cognitive deficits in 51 elderly ILSE participants (baseline age: 63 ± 1 years) who developed MCI or AD 10–12 years later, compared to those who remained cognitively healthy (Frankenberg et al. 2019; Weiner et al. 2019).

MCI is characterized by atrophic processes predominantly affecting temporal lobe regions, including the parahippocampal gyrus, when compared to healthy controls (Dos Santos et al. 2011; Killiany et al. 2002; Pantel et al. 2003). The relationship between language impairment and cerebral atrophy has been explored in several studies. For instance, in a cohort of 50 patients with Alzheimer's disease (AD; Mini‐Mental State Examination, MMSE: 16.8 ± 6.4), naming and praxia were associated with atrophy in the left temporo‐parietal regions, while phonemic verbal fluency correlated with left frontal lobe volume (Pantel et al. 2004). In another study of 24 individuals with MCI/AD (MMSE: 28.8 ± 0.8/23.6 ± 3.9), language impairments—specifically verbal semantic fluency and naming tasks that engage semantic and phonologic processes—were linked to cortical atrophy in the left temporal and parietal lobes, bilateral frontal lobes and right temporal pole (Apostolova et al. 2008). Similarly, research from our group demonstrated that reduced gray matter in the left dorsolateral prefrontal cortex, superior temporal gyrus, and thalamus was associated with impaired semantic verbal fluency (Dos Santos et al. 2011). Deficits in the ‘naming’ subtest of the Consortium to Establish a Registry for Alzheimer's Disease (CERAD) were further associated with atrophy in the bilateral temporal cortex, including the hippocampus. These findings applied to healthy controls (*n* = 32; MMSE: 29.2 ± 0.8), individuals with MCI (*n* = 60; MMSE: 26.4 ± 1.8) and patients with AD (*n* = 34; MMSE: 21.4 ± 2.2). Consistent with these structural findings, fluorodeoxyglucose positron emission tomography (FDG‐PET) studies also reported significant associations between verbal fluency deficits and changes in the left frontal cortex, emphasizing the functional importance of these regions—including Broca's area—for word generation and speech (Desgranges et al. 1998; Welsh et al. 1994).

Based on previous neuroimaging studies of language processing in aging and cognitive decline, we hypothesized that perplexity measures would correlate with gray matter volume in temporal and parietal regions critical for language comprehension and memory integration. Specifically, we anticipated that these associations would reflect the distinct aspects captured by 1‐g and 2‐g perplexity measures, with 1‐g correlating with core language processing regions and 2‐g showing additional correlations with regions supporting autobiographical memory and contextual integration.

## 2 Methods

### 2.1 Participants

The present study utilized participants of the ILSE, a population‐based longitudinal investigation comprising two birth cohorts born in 1930–1932 (C30) and 1950–1952 (C50) (Martin and Martin 2000). To date, the project has completed four examination waves (1993–1996: t1, 1997–1999: t2, 2005–2007: t3, 2013–2016: t4), which comprised medical (including laboratory tests), psychiatric, and psychological assessments (including neuropsychology) and semi‐standardized autobiographical interviews with a duration between 0.5 and 6 h, depending on the time of measurement (see also: Frankenberg et al. 2019). A total of 1002 participants (500 C30 and 502 C50) were randomly recruited from community registers in eastern (Leipzig) and western (Heidelberg) parts of Germany. The study was approved by the ethics committee of the medical faculty of the University of Heidelberg. Written and oral informed consents were obtained from each participant in accordance with the Declaration of Helsinki (1964) and its later amendments after the procedure of the study had been fully explained.

For the present study, a subsample of participants from the C30 cohort at t3 was selected. This subsample included 42 participants who completed all required voice recordings of autobiographical interviews (lasting 0.5 and 2.5 h), neuropsychological data, and MRI scans. Participants were classified as either cognitively healthy or diagnosed with MCI based on the criteria for aging‐associated cognitive decline (AACD) (Levy 1994; Schönknecht et al. 2005). The AACD criteria include both subjective (self‐reported) and objective cognitive impairments, as demonstrated by neuropsychological test performance at least one standard deviation below age‐ and education‐adjusted norms. Affected cognitive domains include memory and learning, attention and concentration, abstract thinking, language, and visuospatial functioning. MCI was diagnosed when an individual met the criteria for AACD and presented no evidence of cerebral or systemic disorder based on history and/or objective examinations. Participants with cognitive deficits attributable to primary physical conditions, such as tumors or cardiovascular disease, were excluded.

Additionally, participants with poor‐quality MRI data (*n* = 4) were excluded. The final sample comprised 38 right‐handed participants, including 26 cognitively healthy controls (HC) and 12 individuals diagnosed with MCI. The demographic characteristics of the sample, along with neuropsychological and perplexity scores, are presented in Table 1.

**TABLE 1:** Sample description.

	All participants (*n* = 38)	Cognitively healthy (*n* = 26)	MCI (*n* = 12)	Test statistic_(df)_	*p* ^a^
Age, in years	73.92 (0.94)	73.85 (0.97)	74.08 (0.90)	*t*_(36)_ = −0.72	0.48
Sex, *n* (male/female)	17/21	12/14	5/7	*χ* ^2^_(1)_ = 0.06	0.80
Education, in years	12.11 (3.22)	13.00 (3.43)	10.17 (1.47)	*t*_(36)_ = 2.73	0.01
Mini Mental State Examination	28.82 (1.16)	28.96 (1.18)	28.50 (1.09)	*t*_(36)_ = 1.15	0.26
Trail making test A	35.25 (10.21)	32.49 (7.83)	41.24 (12.41)	*t*_(36)_ = −2.64	0.01
Trail making test B	96.42 (37.32)	83.98 (24.15)	123.38 (46.90)	*t*_(36)_ = −3.43	0.001
Logical memory—immediate^1^	22.68 (6.63)	25.56 (5.40)	16.67 (4.70)	*t*_(35)_ = 4.88	< 0.001
Logical memory—delayed^2^	18.14 (6.56)	21.46 (5.23)	11.50 (2.71)	*t*_(34)_ = 6.16	< 0.001
1‐g perplexity	197.88 (29.33)	202.01 (32.28)	188.93 (19.92	*t*_(36)_ = 1.29	0.21
2‐g perplexity	114.46 (21.49)	117.59 (23.56)	107.67 (14.80)	*t*_(36)_ = 1.34	0.19

*Note:* Data are means (SDs), unless otherwise indicated; ^1^ *N* = 37; ^2^ *N* = 36; ^a^ *p* values for the difference between cognitively healthy subjects and patients with MCI; MCI: mild cognitive impairment.

### 2.2 Perplexity and Cognitive Assessment

Automatic speech processing has emerged as a promising approach to support cognitive diagnostics. To this end, we developed an automatic speech recognition (ASR) system for linguistic analysis based on the ILSE interviews (Weiner et al. 2017; Weiner et al. 2016). Acoustic and linguistic features were extracted from the interview recordings and their transcriptions, and classifiers were trained on these features. These classifiers demonstrated encouraging results in distinguishing cognitive diagnoses, achieving accurate predictions as early as 12 years prior to the clinical manifestation of MCI or AD (Frankenberg et al. 2019; Weiner et al. 2019).

Perplexity has been identified as a key linguistic feature in evaluating language patterns in spontaneous speech (Weiner et al. 2017), alongside lexical richness (Ablimit et al. 2022). The semi‐standardized autobiographical interviews, conducted across four examination waves, included three parts: open narrative‐generating questions, explicit questioning about defined life events (e.g., elementary school, career entry), and future‐oriented questions about desires and plans. Interview durations ranged from 2.5–6 h during the first wave to 0.5–2.5 h in subsequent waves. Recordings from the first two waves were analog, while those from the latter two waves are digital. All recordings underwent audio alignment and manual transcription, with inter‐rater reliability checks ensuring transcription accuracy. The ASR system, based on a deep neural network trained on 256 h of data, processed these recordings with word error rates ranging from 56.0% to 70.2% across different cognitive groups. Perplexity was calculated using *n*‐gram language models through cross‐validation to assess participants' language patterns. Specifically, 1‐g models analyzed individual words independently of their context, while 2‐g models incorporated the immediate preceding word, providing a contextual approach to text probability modeling (Frankenberg et al. 2019; Weiner et al. 2017; Weiner et al. 2016). This dual approach to perplexity calculation enabled a nuanced analysis of language patterns, offering valuable insights into the cognitive status of participants.

A comprehensive cognitive assessment was performed during each evaluation period, incorporating several standardized neuropsychological instruments. The cognitive battery comprised the MMSE (Folstein et al. 1975), which serves as a widely recognized dementia screening tool from the German version of the CERAD (Aebi 2002; Morris et al. 1989; Welsh et al. 1994). The Trail Making Test (TMT; Reitan 1992) was administered to evaluate information processing speed and executive functioning, where completion time serves as the performance metric, with longer durations indicating lower performance. The logical memory subtest (Wechsler Memory Scale; Petermann and Lepach 2012) was implemented to measure verbal memory capabilities (immediate and delayed).

### 2.3 MRI Data Acquisition

All participants undertook a structural brain scan in a 1.5‐Tesla Siemens scanner (Erlangen, Germany). High‐resolution T1‐weighted structural imaging data were acquired through a three‐dimensional magnetization‐prepared rapid gradient‐echo (MPRAGE) sequence (TR = 10 ms, TE = 4 ms, slice number = 126 coronal slices, image matrix = 256 × 256, voxel size = 0.98 × 0.98 × 1.8 mm).

### 2.4 MRI Data Analysis

The structural MR images underwent preprocessing using CAT 12 (https://neuro‐jena.github.io/cat/) within SPM12 (Wellcome Department of Imaging Neuroscience; http://www.fil.ion.ucl.ac.uk/spm) on the Matlab 2013b platform (http://www.mathworks.com/products/matlab). Voxel‐based morphometry (VBM) analyses were conducted using default parameters: Initially, images were normalized to a Montreal Neurological Institute (MNI) template. Subsequently, the normalized images were segmented into gray matter, white matter, and cerebrospinal fluid compartments using DARTEL‐based techniques, which provide more accurate anatomical alignment across subjects compared to traditional methods (Ashburner 2007; Ashburner and Friston 2000). The resulting gray matter images were smoothed using an 8‐mm full width at half‐maximum Gaussian kernel. Finally, all images were visually checked for artifacts.

To identify cerebral correlates of perplexity in the MRI scans, a sequential data analysis was performed based on the smoothed gray matter images. We first conducted a whole‐brain analysis to identify significant brain regions associated with perplexity (1‐g and 2‐g). Based on these results, we selected corresponding regions from the AAL (Automated Anatomical Labeling) template to define our regions of interest (ROIs) (Rolls et al. 2020). These predefined ROI masks were then applied to limit the analysis to the significant clusters identified in the whole‐brain analysis. By performing the statistical analysis within these specific ROIs, we reduced the number of comparisons, thereby controlling for multiple comparisons and improving the accuracy of our results. This approach allowed us to focus on the most relevant brain areas, ensuring both precision and robustness in the findings.

### 2.5 Statistical Analysis

Demographic and clinical data were analysed using SPSS software version 23; *p* values of less than 0.05 were considered significant. Group differences in demographic and clinical characteristics were investigated using independent group *t*‐tests or *χ* ^2^‐tests. Pearson's correlation coefficients (Pearson's *r*) were calculated to explore potential associations between perplexity and demographical variables and neuropsychological domains, respectively. Bonferroni correction was applied to correct for multiple tests, which resulted in adjusted significance levels.

The gray matter differences between the two groups were compared using a *t* test. Further correlation analyses were conducted in two steps. First, whole‐brain analyses using multiple regression examined the associations between gray matter volume and perplexity measures (1‐g and 2‐g) across all participants, controlling for sex, years of education, and total intracranial volume. Second, region‐of‐interest (ROI) analyses were performed on brain regions showing significant associations in the whole‐brain analysis, using anatomically defined masks from the AAL3 atlas. Both analyses employed a statistical threshold of *p* < 0.001 (uncorrected) at the voxel level with a minimum cluster size of 100 voxels.

## 3 Results

In our analysis, both participants with MCI and cognitively healthy controls were included together, primarily due to the small sample size of the MCI group (*n* = 12), which limited the statistical power of separate group comparisons. Moreover, no significant differences in brain regions were found between the two groups in the gray matter analysis. The prevalence of MCI in our sample (approximately 1/3) is consistent with the rate observed in the general aging population (Schröder and Pantel 2011), supporting the inclusion of both groups in a unified analysis.

Clinical characteristics and neuropsychological performance of subjects are summarized in Table 1. When compared to healthy participants, subjects with MCI had significantly fewer years of education (*t*_(36)_ = 2.73, *p* < 0.01). Additionally, the two groups differed significantly with respect to TMT A/B (*t*_(36)_ = −2.64, *p* < 0.05; *t*_(36)_ = −3.43, *p* < 0.01 respectively) and verbal memory (immediate, *t*_(35)_ = 4.88, *p* < 0.001; delayed, *t*_(34)_ = 6.16, *p* < 0.001), with reduced neuropsychological performance in the MCI group. However, MMSE and perplexity values showed only minor, non‐significant differences between the groups (*p* > 0.10; see Figure S1 for perplexity distribution). After correcting for multiple tests using Bonferroni correction, which resulted in an adjusted significance level of *p* = 0.005, the group differences concerning education and TMT A no longer remained significant.

Correlational analyses revealed that education was significantly associated with delayed logical memory performance (*r* = 0.423, *p* < 0.05) (Table 2). Education showed moderate, non‐significant correlations with other cognitive measures, including TMT‐B (*r* = −0.315, *p* > 0.05). A strong correlation was observed between the two perplexity measures (1‐g and 2‐g, *r* = 0.841, *p* < 0.001), while perplexity showed no significant associations with either education or cognitive measures (all *p* values > 0.05). These patterns remained similar after controlling for education and sex in partial correlation analyses (Table S2).

**TABLE 2:** Correlations between education, cognitive performance, and perplexity measures (Pearson's r).

Variables	1	2	3	4	5	6	7	8	9
1. Education, in years	—								
2. Sex	−0.420**	—							
3. Mini Mental State Examination	−0.089	0.086	—						
4. Trail making test A	−0.166	0.125	−0.352*	—					
5. Trail making test B	−0.315	0.141	−0.419**	0.641***^a^	—				
6. Logical memory—immediate^1^	0.308	−0.052	0.197	−0.234	−0.370*	—			
7. Logical memory—delayed^2^	0.423*	−0.041	0.134	−0.201	−0.307	0.870***^a^	—		
8. 1‐g perplexity	0.044	−0.222	0.134	0.112	−0.106	0.222	0.267	—	
9. 2‐g perplexity	0.028	−0.101	0.142	0.091	−0.087	0.268	0.320	0.841***^a^	—

*Note:* ^1^ *N* = 37; ^2^ *N* = 36; *p < 0.05, **p < 0.01, ***p < 0.001, ^a^significant after Bonferroni correction.

**FIGURE 1:**
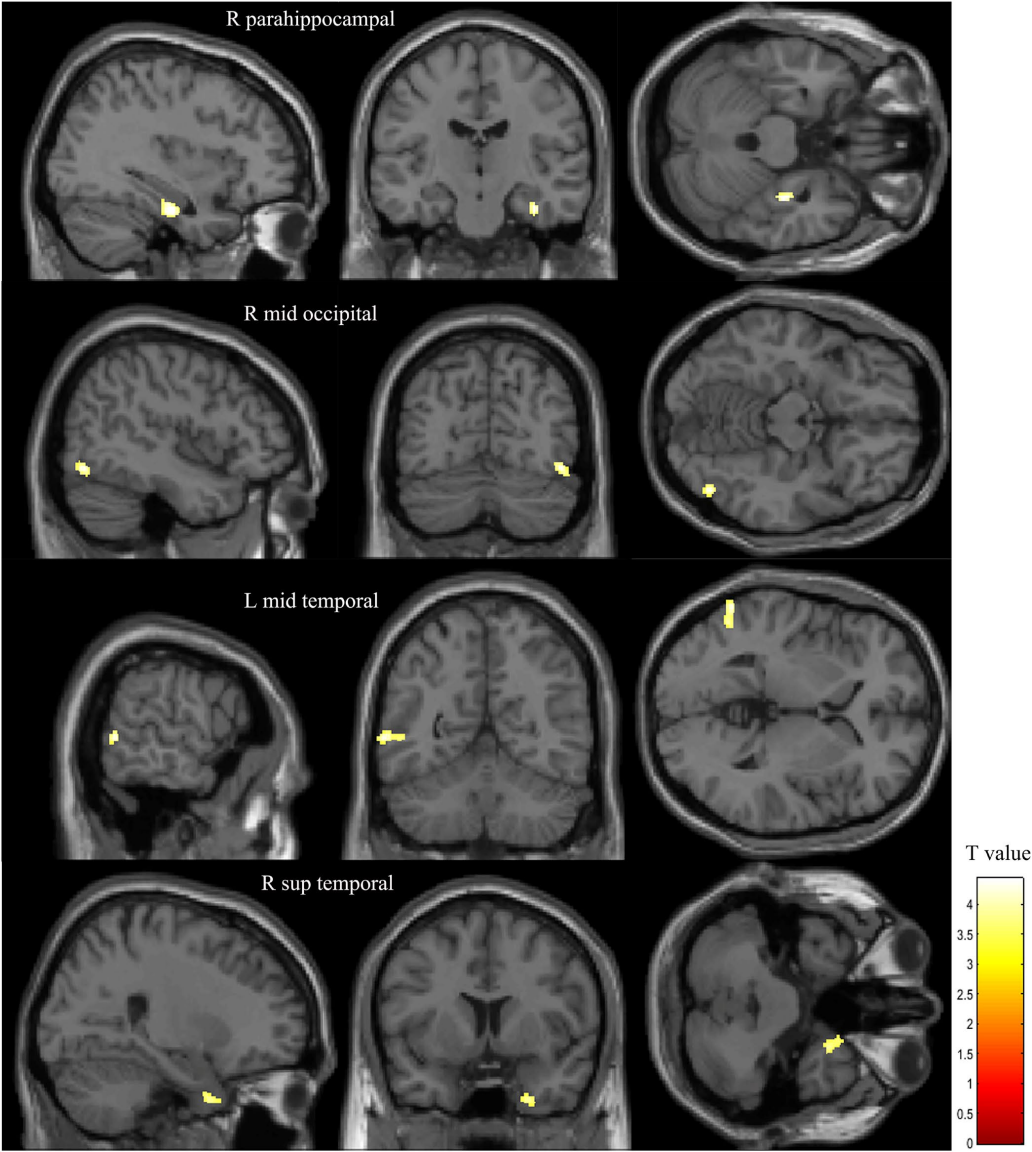
Regions of significant positive correlations between 1‐g perplexity and gray matter volume in participants. Statistical threshold: *p* < 0.001, uncorrected, minimum cluster size = 100 voxels.

**FIGURE 2:**
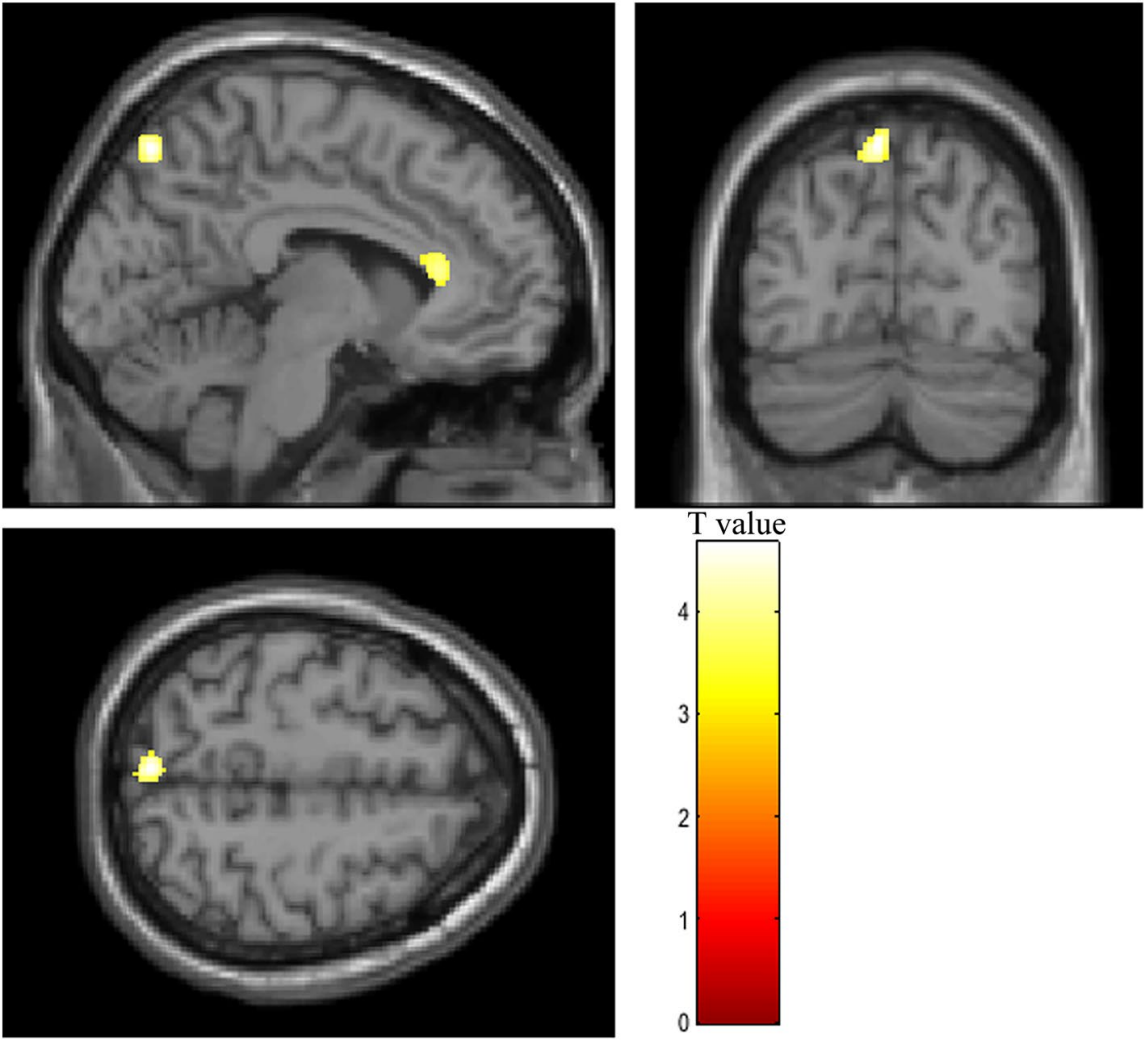
Regions of significant positive correlations between 2‐g perplexity and gray matter volume in participants. Statistical threshold: *p* < 0.001, uncorrected, minimum cluster size = 100 voxels.

Further analyses were performed to examine the associations between gray matter volume and perplexity measures in the whole group. For 1‐g perplexity, significant positive associations with gray matter were found in the right parahippocampal gyrus, right middle occipital gyrus, left middle temporal gyrus, and right superior temporal gyrus. Two‐gram perplexity was positively associated with gray matter in the left precuneus and left anterior cingulate (Table 3, Figures 1 and 2). None of these results remained significant after correction for multiple comparisons (false discovery rate). However, the ROI analyses confirmed significant associations for 1‐g perplexity in the left middle temporal gyrus (*t*‐value = 4.30) and for 2‐g perplexity in the left precuneus (*t*‐value = 4.45) and the left anterior cingulate (*t*‐value = 4.39), with *p <* 0.001 (Table S1).

**TABLE 3:** Anatomical structures showing significant positive correlations between 1‐g/2‐g perplexity and gray matter volume in participants (with sex, years of education and total intracranial volume as covariates).

	Anatomical structures	Cluster size (voxel)	*t*‐value^a^	Peak MNI coordinates x, y, z
1‐g	Right parahippocampal gyrus	104	4.43	36, −17, −26
	Right middle occipital gyrus	109	4.29	47, −72, −14
	Left middle temporal gyrus	129	4.24	−60, −56, 2
	Right superior temporal gyrus	123	3.82	26, 8, −41
2‐g	Left precuneus	217	4.66	−5, −75, 57
	Left anterior cingulate	189	4.09	−8, 29, 12

^a^ *p* < 0.001, uncorrected for multiple comparisons, threshold = 100 voxel.

## 4 Discussion

The present study aimed to investigate the cerebral correlates of perplexity in speech, using extensive autobiographical interviews and structural MRI scans from participants in a population‐based study. Our preliminary analyses suggest that perplexity, as a measure of speech predictability, is associated with a wide network of cerebral regions, including the right parahippocampal gyrus, right middle occipital gyrus, left middle temporal gyrus, right superior temporal gyrus, left precuneus, and left anterior cingulate. However, only the left middle temporal gyrus and left precuneus—regions important for language comprehension and autobiographical memory retrieval—were confirmed as significant in subsequent ROI analyses.

These findings indicate that higher perplexity scores are associated with larger volumes in these brain areas, suggesting that greater variability (and reduced predictability) in speech is associated with better cognitive function. This result aligns with previous work, such as a functional MRI study by Lopopolo et al. (2017), which found that perplexity at varying levels of abstraction predicted activity in the temporal, inferior parietal, and perisylvian cortices in healthy participants listening to naturalistic linguistic input.

Language changes are known to emerge during the course of AD, with significant impacts on communication abilities. These changes, which affect speech production (speech rate), syntactic (length of utterance), lexical (word‐frequency, use of pronouns), fluency (repetitions, word‐finding difficulties), and semantics (information units), have profound functional consequences for daily life (Slegers et al. 2018). Perplexity measures, which capture variability in speech and its predictability, may thus provide valuable insight into these language changes over the course of cognitive decline. Moreover, reduced perplexity may serve as an early indicator of cognitive decline, potentially appearing years before the clinical onset of MCI/AD (Frankenberg et al. 2019).

Although perplexity scores were significantly intercorrelated in our sample, the overall non‐significant correlation between perplexity and traditional neuropsychological domains suggests that perplexity may represent an additional cognitive feature, capturing unique aspects of language processing. A previous study from our group (Frankenberg et al. 2019) also revealed only minor, non‐significant associations between perplexity measures and neuropsychological performance in healthy elderly individuals. These findings support the hypothesis that perplexity goes beyond traditional neuropsychological domains, as it measures properties of language. This points to a distinct relationship between language deficits and memory decline in MCI (McCullough et al. 2019; Sherman et al. 2021).

Notably, in our structural imaging analyses, perplexity—as a measure of linguistic complexity (Frankenberg et al. 2019)—showed associations with specific brain regions involved in language processing and memory. These findings suggest that perplexity could be sensitive to subtle linguistic changes. While such changes might not yet be detectable by structural neuroimaging or traditional cognitive measures like the MMSE at the early stages of cognitive decline, perplexity may capture cognitive and language‐related deficits that traditional screening tools fail to identify (McCullough et al. 2019; Sherman et al. 2021). Given the absence of significant structural differences between MCI and HC, we combined both groups to explore these brain‐language relationships, though this approach limits our ability to draw definitive conclusions about the diagnostic utility of perplexity.

The lower education level in the MCI group, in contrast to the cognitively healthy participants, aligns with previous research demonstrating that education acts as a key component of cognitive reserve, helping to protect against MCI/AD (Sattler et al. 2012; Stern 2009). The neurocognitive profile of our MCI group showed characteristic impairments in verbal memory, information processing speed, and executive functioning, as anticipated. However, the MMSE, as a dementia screening tool, revealed comparable scores between the groups (Andrejeva et al. 2016). The same pattern applied to perplexity values.

Language processing involves a distributed neural network (Geranmayeh et al. 2014), and our exploratory analyses revealed several regions associated with perplexity. The whole‐brain analysis identified associations with the right parahippocampal gyrus, which is crucial for memory encoding and retrieval (Eichenbaum et al. 2007), the right middle occipital gyrus, involved in visual word processing (Price 2012), the right superior temporal gyrus, supporting speech perception (Hickok and Poeppel 2007), and the left anterior cingulate, implicated in speech monitoring (Christoffels et al. 2007). Among these regions, subsequent ROI analyses specifically confirmed the involvement of the left middle temporal gyrus and left precuneus. This distributed pattern reflects the complex nature of spontaneous speech during autobiographical interviews, where multiple cognitive processes, including memory retrieval, language production, and semantic processing, are simultaneously engaged (Bookheimer 2002; Martin 2003). The particular involvement of temporal and parietal structures aligns with their established roles in semantic processing and memory integration, which are essential components of natural speech production.

The middle temporal gyrus, as an integration hub for semantic and phonological functions, is considered highly relevant for language processing (Turken and Dronkers 2011), particularly for lexical‐semantic and conceptual aspects of meaning (Lau et al. 2008; Patterson et al. 2007), as well as for sentence comprehension (Price 2010). In fact, verbal semantic memory (e.g., naming) has been significantly correlated with regional cerebral glucose metabolism in the left middle temporal gyrus in a group of 57 patients with mild AD (Hirono et al. 2001). Similarly, strong correlations between naming performance and gray matter atrophy in the posterior middle and inferior temporal gyri were observed in a group of 24 patients with MCI/AD (Apostolova et al. 2008). These findings are consistent with the association between perplexity and left middle temporal gyrus volume found in the present study.

The precuneus is involved in motor coordination, visuo‐spatial imagery, episodic (autobiographical) memory retrieval, and self‐processing operations, such as first‐person perspective‐taking, and it is connected to the cingulate (Cavanna and Trimble 2006). It has been shown to be activated bilaterally during continuous speech in healthy adults (Kircher et al. 2004). More specifically, semantic processing of spoken words and sentence comprehension were highlighted with respect to precuneus activation (Price 2010). In the present study, these findings were confirmed and extended to perplexity as an indicator of speech predictability and content complexity based on extensive autobiographical interviews. The specific association between 2‐g perplexity and precuneus volume may reflect the increased demands of processing word sequences in context, as this model considers word‐pair relationships rather than isolated words (Frankenberg et al. 2019). This aligns with the precuneus' role in integrating temporal information (Cavanna and Trimble 2006; Price 2010) during autobiographical speech production (Kircher et al. 2004).

Taken together, the correlations between perplexity and volumes of the left middle temporal gyrus and left precuneus, as confirmed in ROI analyses, emphasize the heterogeneity of perplexity. More specifically, perplexity refers not only to functional aspects of language, reflected by its association with the middle temporal gyrus, but also to content‐related aspects, such as autobiographical memory retrieval. The latter is reflected by its association with the precuneus and is further supported by the fact that perplexity was obtained from extensive autobiographical interviews.

The small sample size of the present study limits the generalizability of our results. However, as our sample is part of an age cohort, the homogeneity of age rules out an age‐related influence on the results. Additionally, replication of our findings in data from other languages is necessary, as language‐specific characteristics (e.g., word order) could affect measures of perplexity. We controlled for potential confounders such as total intracranial volume, sex, and education in our analyses. Furthermore, the effect of concomitant physical diseases on cognition (e.g., mild cognitive impairment) was excluded due to our strict inclusion criteria. Of the 38 participants, 12 were diagnosed with MCI, a condition frequently observed in the seventh decade of life, with prevalence rates of approximately one‐third in this age group (Schröder and Pantel 2011). Given that our results are uncorrected for multiple comparisons, they should be considered preliminary. Further research with larger samples and/or alternative methods such as network analyses is needed to substantiate our findings.

## 5 Conclusion

In conclusion, our exploratory analyses revealed distinct associations between perplexity measures and language‐related cerebral areas, particularly the left middle temporal gyrus and left precuneus. These findings provide initial insights into the neural substrates underlying perplexity as a measure of language complexity in aging. While our results should be interpreted cautiously given the lack of significant group differences and the need for multiple comparisons correction, they contribute to understanding the neural basis of language production in aging. Future longitudinal studies with larger samples are needed to determine whether perplexity and its neural correlates could serve as meaningful indicators of cognitive change over time.

## Conflicts of Interest

The authors declare no conflicts of interest.

## Supporting information


**Data S1.** Supporting Information.

## Data Availability

All relevant data are within the manuscript. Additional data are available from the corresponding authors upon request.
