# Supplementary material for: Structural Cerebral Correlates of Perplexity: Exploring a Linguistic Marker in Cognitive Aging
Source: Psych J. 2025 Mar 14;14(3):407–16. doi: 10.1002/pchj.70010 (PMC12133234; doi:10.1002/pchj.70010)

Table S1. Anatomical structures (6 mm smooth) showing significant positive correlations between 1-gram/2-gram perplexity and grey matter volume in the whole group (with sex, years of education and total intracranial volume as covariates)

|  | Anatomical structures | Cluster size (voxel) | t-value^+^ | Peak MNI coordinates x, y, z |
| --- | --- | --- | --- | --- |
| 1-gram | left middle temporal gyrus | 121 | 4.3 | -62 -56 3 |
| 2-gram | left precuneus | 145 | 4.45 | -6 -74 56 |
|  | left anterior cingulate | 203 | 4.39 | -8 30 14 |

^+^*p* < 0.001, uncorrected for multiple comparisons, threshold =100 voxel

Table S2. Correlations between cognitive performance and perplexity measures after controlling for education and sex in the whole sample (Partial correlations)

| Variables | 1 | 2 | 3 | 4 | 5 | 6 | 7 |
| --- | --- | --- | --- | --- | --- | --- | --- |
| 1. Mini Mental State Examination | - |  |  |  |  |  |  |
| 2. Trail making test A | -0.378* | - |  |  |  |  |  |
| 3. Trail making test B | -0.474** | 0.629***† | - |  |  |  |  |
| 4. Logical memory – immediate^1^ | 0.234 | -0.199 | -0.301 | - |  |  |  |
| 5. Logical memory – delayed^2^ | 0.158 | -0.193 | -0.213 | 0.843***† | - |  |  |
| 6. 1-gram perplexity | 0.155 | 0.139 | -0.098 | 0.250 | 0.367* | - |  |
| 7. 2-gram perplexity | 0.152 | 0.104 | -0.082 | 0.289 | 0.379* | 0.845***† | - |

*Note.* ^1^ N=37; ^2^ N=36; * *p* <0 .05, ** *p* < 0.01, *** *p* <0 .001, † significant after Bonferroni correction.

| Variables | 1 | 2 | 3 | 4 | 5 | 6 | 7 |
| --- | --- | --- | --- | --- | --- | --- | --- |
| 1. Mini Mental State Examination | - |  |  |  |  |  |  |
| 2. Trail making test A | -0.08 | - |  |  |  |  |  |
| 3. Trail making test B | -0.422 | 0.372 | - |  |  |  |  |
| 4. Logical memory – immediate | 0.249 | -0.087 | 0.035 | - |  |  |  |
| 5. Logical memory – delayed | -0.01 | -0.159 | 0.296 | 0.684* | - |  |  |
| 6. 1-gram perplexity | 0.53 | 0.39 | -0.35 | -0.351 | -0.553 | - |  |
| 7. 2-gram perplexity | 0.452 | 0.268 | -0.381 | -0.351 | -0.12 | 0.711** | - |

Table S3. Correlations between cognitive performance and perplexity measures after controlling for education and sex in the MCI group (Partial correlations)

*Note.* * *p* <0 .05, ** *p* < 0.01.

Table S4. Correlations between cognitive performance and perplexity measures after controlling for education and sex in the HC group (Partial correlations)

| Variables | 1 | 2 | 3 | 4 | 5 | 6 | 7 |
| --- | --- | --- | --- | --- | --- | --- | --- |
| 1. Mini Mental State Examination | - |  |  |  |  |  |  |
| 2. Trail making test A | -0.485* | - |  |  |  |  |  |
| 3. Trail making test B | -0.418* | 0.721***† | - |  |  |  |  |
| 4. Logical memory – immediate^1^ | 0.078 | 0.1 | -0.154 | - |  |  |  |
| 5. Logical memory – delayed^2^ | 0.016 | 0.304 | 0.14 | 0.796***† | - |  |  |
| 6. 1-gram perplexity | 0.007 | 0.255 | 0.17 | 0.212 | 0.362 | - |  |
| 7. 2-gram perplexity | 0.031 | 0.209 | 0.182 | 0.263 | 0.325 | 0.856***† | - |

*Note.* ^1^ N=25; ^2^ N=24; * *p* <0 .05, *** *p* <0 .001, † significant after Bonferroni correction.

Figure S1. Distribution of perplexity scores in HC and MCI groups


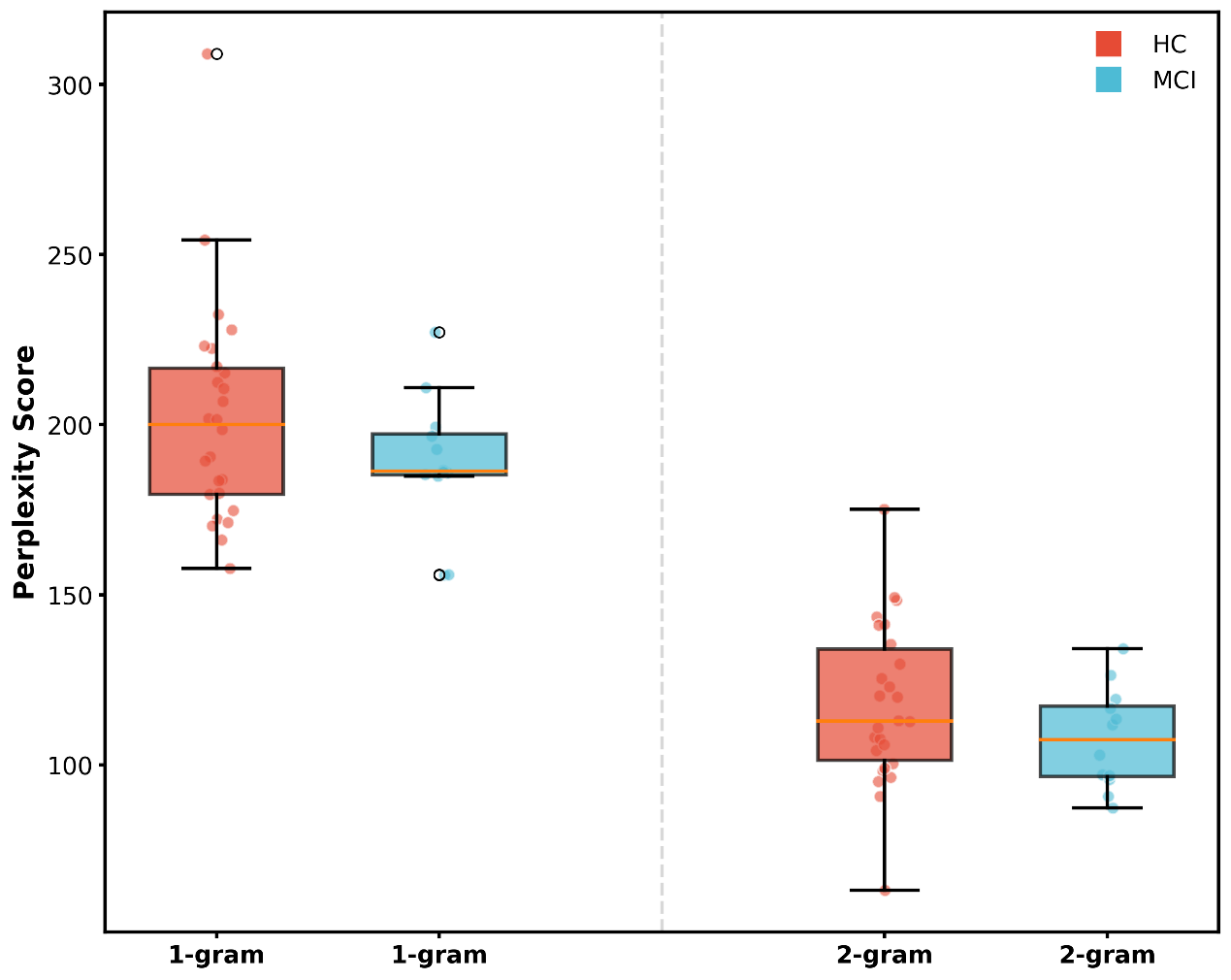

Supplement: Supplementary file 1 — Data S1. Supporting Information. [file PCHJ-14-407-s001.docx]
